# Thyroid function: a new road to understanding age-related macular degeneration?

**DOI:** 10.1186/s12916-015-0343-2

**Published:** 2015-04-23

**Authors:** Till Ittermann, Clemens Jürgens

**Affiliations:** Institute for Community Medicine, University Medicine Greifswald, Walther Rathenau Str. 48, D-17475 Greifswald, Germany

**Keywords:** Thyroid hormone, Thyroid function, AMD, Age-related macular degeneration

## Abstract

Age-related macular degeneration (AMD) continues to be amongst the leading causes of blindness and visual impairment worldwide. AMD remains a degenerative disorder of unknown etiology with rising prevalence. It induces retinal changes and damages those parts of the retina which are essential for central vision. The risk of developing this condition is associated with increasing age. Early stages usually progress without warning signs over years. The major identified risk factors for AMD development are age, ethnicity, family history, and current smoking. Associations of other modifiable risk factors with AMD have been widely published but these studies have reported conflicting results and showed a lack of consistency. According to recent data published in *BMC Medicine* from the population-based Rotterdam study, thyroid hormones may contribute to a better characterization of AMD in clinical practice. In that study serum free thyroxine levels were positively associated with development of AMD. More studies are needed to validate these findings and to understand better the role of thyroid hormones in the pathogenesis of AMD disease.

Please see related article: http://dx.doi.org/10.1186/s12916-015-0329-0

## Introduction

In 1990 and 2010, age-related macular degeneration (AMD) was amongst the leading causes of blindness and visual impairment worldwide [1]. The greatest prevalence was reported in developed countries, especially in high-income regions. Such regions face the strongest demographic shift where overaging societies increase the prevalence of age related diseases in general. In the United States the number of persons affected by AMD was estimated to increase by 50% until 2020, based on population demographics [2]. The visual impairment caused by AMD presents a tremendous economic burden and major limitations in quality of life [3,4].

### Pathophysiology and clinical aspects of AMD

AMD increases strongly with age and is characterized by the presence of drusen or pseudodrusen, abnormalities in retinal pigmentation, geographic atrophy, neovascularization, or angiomatous proliferation. Drusen are small white or yellowish extracellular deposits in the retina. They are located between the retinal pigment epithelium (RPE) and the Bruch’s membrane. A number of grading schemes has been developed based on the size and number of drusen and the amount of other retinal lesions. A common classification was defined by the Age-Related Eye Disease Study (AREDS) [5], which subdivides AMD into the categories no AMD, early AMD, intermediate AMD, and advanced AMD. These stages represent the natural history of AMD, which generally progresses from the ‘dry’ (atrophic) to the ‘wet’ (neovascular) form. It is clinically important that early stages may persist over many years without any signs or discomfort. The typical AMD patient is over 55 years old and often presents with neovascular conditions as the first manifestation. This can be explained by the fact that pathogenic mechanisms of AMD development and progression remain poorly understood. For this reason, there is no causative therapy available. Instead, the rationale for treatment is to prevent vision loss by delaying onset or progression to later stages, and by reducing neovascularization [6]. Treatment modalities have to be adapted to their respective stages, whereas no benefit of therapeutic interventions has been published for early AMD. The conversion from intermediate to advanced AMD might be attenuated by a daily antioxidant therapy. In 2001 the AREDS group reported a reduced odds ratio of 0.72 in the antioxidants group [5]. The introduction of substances that inhibit vascular endothelial growth factors (VEGF) led to more effective treatments of neovascularization. Consequently, intravitreal injection of anti-VEGF drugs has become primary therapy for most cases of advanced AMD [7]. The repeated application of these substances obtains beneficial therapeutic effects in the macular region resulting in good visual restoration. Potential systemic side effects of these agents aroused concerns about cardiovascular and hematologic complications, but the real evidence of such events is largely unknown.

### Screening and diagnostic procedures

Several diagnostic tests have been established to assess signs of AMD. Funduscopy, optical coherence tomography, and angiography are medical imaging procedures used to diagnose AMD and to determine disease progression. Testing visual acuity is essential to evaluate visual impairment. Early treatment is crucial for patients who develop wet AMD, because it reduces the risk of vision loss and may improve the final visual outcome. With the introduction of new therapeutic methods, early detection will also become increasingly important for early AMD stages. However, screening for AMD is not common yet. Although patients’ self-testings (that is, Amsler grid [8]) show low sensitivity [9], best practice guidelines recommend encouraging patients to assess their own visual acuity [6]. Improved knowledge of risk factors may help to develop comprehensive screening strategies.

### Risk factors

Several studies have identified age, ethnicity, and family history (genetics) as major risk factors for the development of wet AMD [10,11]. Smoking is also strongly associated with AMD. It appears to be the only modifiable risk factor that has been consistently identified in the literature [12]. Smoking cessation reduces the risk of AMD development and progression. Thus, it should be beneficial to generate awareness about the risk of developing AMD from the toxic effects of cigarette smoking. Associations with other modifiable risk factors have been widely published but these studies have reported conflicting results and showed a lack of consistency: hypertension and cardiovascular risk factors [13-20], dietary fat intake [11,20-25], aspirin use [26,27], sunlight exposure [28-30], and alcohol [31]. Thus, the pathogenesis of AMD is currently poorly understood and it is important to identify potential risk factors for this disease. The work by Chaker *et al*. suggests that thyroid function is an important risk factor for AMD [32]. In that study, based on data from the population-based Rotterdam study, serum free thyroxine levels were positively associated with development of AMD. There was no association of serum thyroid-stimulating hormone (TSH) with this outcome.

### Association between serum TSH levels and macular degeneration within the Study of Health in Pomerania (SHIP-Trend)

In SHIP-Trend, a population-based cross-sectional study conducted in Northeast Germany, data from 3,070 individuals 20- to 80-years old were available to investigate the association between serum TSH levels and macular degeneration [33]. Serum TSH levels were measured by an immunochemiluminescent procedure (Vista, Siemens, Eschborn, Germany). Macular degeneration was evaluated by an experienced reader based on images from fundus photography of the central retina derived from a non-mydriatic fundus camera (TRC-NW 200, Topcon Corporation, Tokyo, Japan) [34]. There were 62 individuals with macular degeneration in SHIP-Trend (2.0%). In logistic regression adjusted for age, sex, smoking status, hypertension, cholesterol, diabetes and body mass index we found, in agreement with the findings of Chaker *et al*., no significant association between serum TSH levels and macular degeneration (odds ratio (OR) = 0.99; 95% confidence interval (CI) = 0.92 to 1.07). Likewise, there was no significant association between serum TSH levels within the reference range and macular degeneration (OR = 1.08; 95%CI: 0.63 to 1.84). Restricting the age range to individuals ≥55 years did not change the results significantly. Unfortunately, serum fT4 levels were not measured in SHIP-Trend, so that we were not able to verify the significant positive association between fT4 and macular degeneration detected in the Rotterdam study.

## Conclusions

AMD is a disease whose risk factors and pathophysiology are not well understood and, thus, AMD is often diagnosed in an already advanced stage. Thyroid hormones may contribute to a better characterization of AMD in clinical practice as shown by the data of Chaker *et al*. [32].

Even though observational studies cannot determine causal inference, they provide useful evidence to improve the perception of specific illnesses. However, more studies are needed to validate the findings of Chaker *et al*. and to understand better the role of thyroid hormones in the pathogenesis of the AMD disease.
